# Workload in Norwegian general practice 2018 – an observational study

**DOI:** 10.1186/s12913-019-4283-y

**Published:** 2019-06-28

**Authors:** Tone Morken, Ingrid Keilegavlen Rebnord, Kjell Maartmann-Moe, Steinar Hunskaar

**Affiliations:** 1 National Centre for Emergency Primary Health Care, NORCE Norwegian Research Centre, Kalfarveien 31, NO-5018 Bergen, Norway; 2 The Norwegian Directory of Health, Oslo, Norway; 3 Department of Global Public Health and Primary Care, University of Bergen, Bergen, Norway

**Keywords:** General practice, Family medicine, Workload, Survey, Observational study

## Abstract

**Background:**

Rising workload in general practice has been a recent cause for concern in several countries; this is also the case in Norway. Long working hours and heavy workload seem to affect recruitment and retention of regular general practitioners (RGPs). We investigated Norwegian RGPs’ workload in terms of time used on patient-related office work, administrative work, municipality tasks and other professional activities in relation to RGPs, and gender, age, employment status and size of municipality.

**Methods:**

In early 2018, an electronic survey was sent to all 4716 RGPs in Norway. In addition to demographic background, the RGP reported minutes per day used on various tasks in the RGP practice prospectively during 1 week. Working time also included additional tasks in the municipality, other professional work and on out-of-hours primary health care. Differences were analysed by chi square test, independent t-tests, and one-way ANOVA.

**Results:**

Among 1876 RGPs (39.8%), the mean total working hours per week was 55.6, while the mean for regular number of working hours was 49.0 h weekly. Men worked 1.5 h more than women (49.7 vs. 48.2 h, *p* = 0.010). Self-employed RGPs work more than salaried RGPs (49.3 vs. 42.5 h, *p* < 0.001), and RGPs age 55–64 years worked more than RGPs at age 30–39 (51.1 vs. 47.3 h, *p* < 0.001). 54.1% of the regular working hours was used on face-to-face patient work.

**Conclusions:**

Norwegian RGPs have long working hours compared to recommended regular working hours in Norway, with small gender differences. Only half of the working time is used on face-to-face consultations. There seems to be a trend of increasing workload among Norwegian GPs, at the cost of direct patient contact. Further research should address identifying factors that can reduce long working hours.

**Electronic supplementary material:**

The online version of this article (10.1186/s12913-019-4283-y) contains supplementary material, which is available to authorized users.

## Background

In recent years there have been increasing concerns in several countries about the rising workload in general practice. Several changes that affect the workload of general practitioners (GPs) include more multimorbidity, an ageing population, increased patient demand, pressure to reduce access to secondary care, growing number of responsibilities, escalating administrative tasks, and more documentation requirements [1–5]. A systematic literature review showed that decreased job satisfaction was associated with long working hours, administrative burdens, heavy workload and lack of time [6]. Rising workload in general practice seems to challenge both recruitment and retention of GPs [1, 7, 8].

In Norway, a national regular general practitioner (RGP) scheme was implemented in 2001, aiming to promote continuity in the doctor-patient relationship [9, 10]. All inhabitants who are registered in the National Registry as living in Norway are assigned to or choose an RGP, and the RGPs are gatekeepers to all specialties [9, 11]. Primary health care including RGPs is the responsibility of the municipalities, and each RGP has therefore a contract with the municipality. The number of inhabitants in each municipality varies from a few hundred to nearly 700,000. In addition to standard patient consultations, the RGPs can be required to perform other RGP tasks 1 day per week, for example in parent and child clinics, youth health services, or in nursing homes. In addition to the regular working hours, the RGPs are responsible for participating in out-of-hours (OOH) primary health care.

An ongoing recruitment challenge in general practice in several countries has resulted in an ageing RGP population, partly explained by too many new tasks and insufficient framework conditions [1–3, 7, 12–14]. The recruitment challenges seem to be an increasing problem, not only in rural areas as previously reported [15], but in urban areas as well, resulting in increasing numbers of vacancies and short time locums. In Norway, several stakeholders have presented an interest in updated data on workload in general practice. One study found that the weekly total hours constituted 46.4 h [16], while in 2014 it was 48.6 [17]. However, little research has assessed workload by time and job content, and there is need for an updated, representative and more detailed knowledge about regular working hours. Therefore, we investigated the RGPs’ workload in terms of time used on patient-related office work, administrative work, municipality tasks and other professional activities. We also examined the differences in working hours by gender, age, employment status, size of patient list and size of municipality.

## Methods

### Setting and design

An electronic questionnaire made for this study (Additional file 1) was sent by email to all available RGPs (*n* = 4716) in Norway. The purpose was to monitor working hours of RGPs as precisely as possible during 1 week in January 2018. The mailing list was based on addresses from Norwegian Healthnet (NHN) and The Norwegian Health Economics Administration (Helfo). Non-responders received a reminder email one and 2 weeks after the first email. In addition to the invitation email, the Norwegian Directorate of Health sent information about the study to all municipalities, and The Norwegian Medical Association sent information to all their RGP members, in order to encourage RGPs to participate in the study. The study protocol was submitted to and approved by the Ombudsman for Research, Norwegian Centre for Research Data (NSD).

Data on demographic background of the RGP population in Norway was collected from the Norwegian Directorate of Health [18]. A technical report with preliminary results from the survey was delivered to the Norwegian Directorate of Health [19]. The report was used by the Ministry of Health, the Norwegian Directorate of Health, The Norwegian Medical Association and Norwegian Association of Local and Regional Authorities in policy making and negotiations for new contracts and regulations.

### Survey instrument

The authors designed the questionnaire using Qualtrics software (version 2018 of Qualtrics, copyright© 2018, Provo, UT) and pilot-tested it on 30 RGPs. The questionnaire included the following items: Gender, age (categorised), number of inhabitants on list of the RGP (categorised), and number of inhabitants in the municipality (categorised). For each of the 7 days during 1 week, the RGP should report minutes per day used on various tasks in their patient-related office practice, such as patient consultations, clinical meetings, home visits, referrals, certificates, telephone contacts and e-consultations. Working time questions also included time on additional tasks in the municipality (parent and child clinic, youth health services, nursing home, conferences/supervision), other professional work, and on OOH primary health care.

### Statistical analyses

In the analyses, the category ‘face-to-face patient work’ included patient consultations, clinical meetings, and home visits. Additional tasks in the community or out-of hours primary health care were not included in the category. The category ‘other patient-related work’ included writing records notes, referrals and certificates, telephone contacts and e-consultations. We used descriptive statistics given as mean, standard deviation (SD) and proportions. To identify statistically significant differences between groups, we used Pearson chi square test, independent t-test, and one-way ANOVA, with Bonferroni correction for post hoc tests. The level of statistical significance was set at *p* = 0.05. The statistical analyses were performed using IBM SPSS Statistics version 25.0.

## Results

Among the 4716 RGPs, 1955 (41.4%) responded. Of them, we excluded 79 persons as they reported that they did not work as RGPs or were on sick leave at the time of the study. 1876 RGPs (39.8%) were thus included in the final analyses.

### Demographic data

Table 1 shows the demographic characteristics of the RGPs in the study sample compared with the general RGP population in Norway [18]. The gender and age distribution as well as patient list lengths were similar. Both in the sample and in the Norwegian RGP population, most RGPs were specialists in general practice, and women had shorter patient lists than men (*p* < 0.001). Women were significantly younger than men (*p* < 0.001).

**Table 1:** Demographic characteristics of the regular general practitioners (RGPs) (*n* = 1876) N/A: Data not available

Characteristics		Study population		Total Norwegian RGP population
		n	%	%
Woman		910	48.5	42.0
Age				
	< 30 years	49	2.6	2.0
	30–39 years	589	31.4	27.8
	40–54 years	705	37.6	37.8
	55–64 years	429	22.9	28.4
	≥ 65 years	89	4.7	3.9
Experience as GP				
	0–10 years	852	45.4	N/A
	11–25 years	563	30.0	N/A
	> 25 years	430	22.9	N/A
GP specialist		1267	67.5	61.8
Locum doctor		108	5.8	18.6
Employment position				
	Self-employed	1776	94.6	93.4
	Fixed salaried	73	3.9	6.6
Size of patient list				
	≤ 600	72	3.8	6.2
	601–900	316	16.8	18.4
	901–1200	721	38.4	38.3
	1201–1500	567	30.2	26.4
	1501–1800	139	7.4	7.0
	≥ 1800	43	2.3	3.6
Inhabitants in municipality of practice				
	≤ 10,000	318	17.0	N/A
	10,001–25,000	407	21.7	N/A
	25,001–100,000	657	35.5	N/A
	> 100,000	471	25.1	N/A

### Additional position in the municipality and other professional activities

828 RGPs (44.1%) had one or more positions in the municipality in addition to the RGP consultation practice. The most common additional position in the municipality was being a doctor at a mother and child clinic (22%) (Table 2). The share of RGPs that had additional positions in the municipality was higher in the small municipalities with 10,000 inhabitants or less (86.0% vs 43.1% of RGPs, *p* < 0.001) among younger RGPs (57.1% among age below 30 vs 30.3% among age 65 years or above *p* < 0.001), among non-specialists (49.5% vs 41.8%, *p* = 0.002) and among RGPs with fixed salary (71.2% vs 43.1%, *p* < 0.001). 640 physicians (34.1%) participated in OOH primary care during the week of registration.

**Table 2:** Participation and mean hours per week for work tasks in addition to regular general practitioner consultation practice (*n* = 1876) N/A: Data not available

	Physicians		Hours per week
	n	%^a^	Mean
Parent and child clinic	414	22.1	5.1
Nursing home/elder care	269	14.3	7.7
Administrative position (district medical officer, casualty clinic medical officer, infection control, adviser)	204	10.9	8.6
Youth health services	90	4.8	4.8
Municipal emergency beds	57	3.0	5.1
Day time position at casualty clinic with fixed salary	45	2.4	6.6
Other patient work (prison etc.)	65	3.5	6.4
No additional position in the municipality	1051	56.0	N/A
Other non-municipality positions (research, education, consultant etc.)	411	21.9	8.7

^a^The total percent is more than 100 as some physicians had more than one additional position in the municipality

279 RGPs (14.9%) had both a position in the municipality and other professional activities in addition to the RGP consultation practice. 76 RGPs (4%) had other professional activities like research/education or being liaison with local hospital, with no additional position in the municipality.

### Total working hours per week

**Fig. 1:**
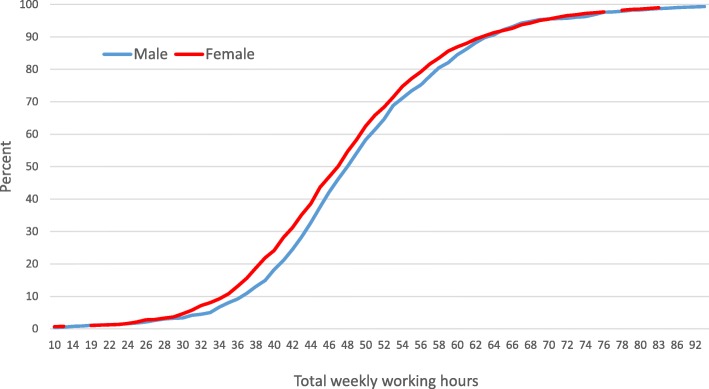
Total weekly working regular hours and cumulative percent by gender (*n* = 1876)

The total number of hours worked per week was on average 55.6 (SD 20.3, median 52.5). Further results are presented based on regular working hours, defined as total working hours minus hours on OOH primary care. Table 3 shows regular working hours per week and by different tasks. The regular working hours was on average 49.0 (SD 12.4, median 48.0). 79.8% of the RGPs worked more than 40 h and 10% worked more than 63 regular working hours per week. Fig. 1 shows the total weekly regular working hours and cumulative percent of men and women. Men worked on average 49.7 h (SD 12.2) and significantly more than women, who worked on average 48.2 h (SD 12.5) (t-test 95% CI = 0.36–2.60, *p* = 0.010). The self-employed RGPs worked on average 7 h more per week than the RGPs with fixed salary (49.3 vs. 42.5 h, t-test 95% CI = 3.93–9.65, *p* < 0.001). RGPs in the 55–64 age category worked the highest number of hours per week (51.1 h) and significantly more than the 30–39 age category (47.3 h, one-way ANOVA with post hoc Bonferroni, 95% CI = 1.54–5.92, *p* < 0.001) and the 65 years and above age category (46.4 h, 95% CI = 0.68–8.71, *p* = 0.010). RGPs in the largest municipalities (> 100,000 inhabitants) worked significantly more regular working hours per week (50.8 h) than RGPs in the smallest municipalities (< 10,000 inhabitants) (44.37 h, one-way ANOVA with post-hoc Bonferroni, 95% CI = 1.57–11.22).

**Table 3:** Hours per week on different patient-related office work, additional positions in the community and on other professional activities (*n* = 1876)

	Hours per week						Mean proportion of total regular working hours
	Percentiles						%
	10	25	50	75	90		
Total patient-related office work (*n* = 1876)	25.8	32.1	38.4	45.1	51.6		79.7
Face-to-face consultations							
Face-to-face consultations at the office	16.5	20.5	24.8	28.8	32.8		51.0
Clinical meetings	0.0	0.0	0.5	1.5	2.8		2.0
Home visits	0.0	0.0	0.0	1.0	2.0		1.1
Other patient related work							
Medical records without patient present	2.8	4.3	6.5	9.5	12.8		14.7
Certificates and declaration	0.0	0.5	1.5	2.8	4.3		3.9
Phone/e-mail patient/next of kin	0.5	1.0	1.5	2.4	3.5		3.7
Phone/e-mail others	0.3	0.8	1.1	1.8	2.6		2.7
E-consultations	0.0	0.0	0.0	0.0	1.3		0.6
Additional community position (*n* = 828)	2.5	3.5	5.5	7.5	10.5		13.0
Other professional activities (*n* = 411)	0.0	0.0	0.0	9.0	15.6		8.5
Administration of office practice (*n* = 1876)	0.0	0.8	2.0	3.8	6.5		5.0
Total regular working hours (*n* = 1876)	35.8	41.6	48.0	55.4	63.3		100.0

### Working hours on different tasks

**Fig. 2:**
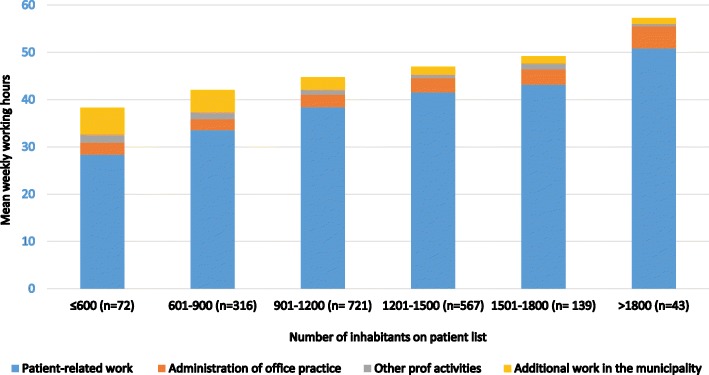
Mean weekly regular working hours by different working tasks, by size of patient list (*n* = 1876)

The RGPs worked on average 9.5 h per day (SD 2.8) and 38.8 h per week with patient-related office work including both “face-to-face consultations” and other patient-related tasks, which is 79.2% of the total weekly regular working hours (Table 3). The face-to-face consultations (at the office, home visits and clinical meetings) accounted for 54.1% of the total weekly regular working hours. Administration of the RGP practice accounted for 5% of the total regular working hours per week. Fig. 2 shows the mean weekly regular working hours on different tasks by size of patient list. RGPs with larger patient lists had more hours of patient-related work (*p* < 0.001), more hours of administration practice (*p* < 0.001), while RGPs with smaller patient lists had more hours of additional tasks in the municipality (*p* < 0.001) and other professional activities (*p* = 0.047).

The 828 (44.1%) RGPs that had additional positions in the municipality, worked on average 6.1 h per week on municipality tasks. The 411 (21.9%) RPGs that had other additional professional activities, worked on average 1 h per week on those activities. The RGPs in the smallest municipalities (≤10,000 inhabitants) had significantly more hours of additional work in the municipalities than the others (*p* < 0.001).

### Discussion

This study shows that the RGPs in Norway have a high number of regular working hours, with small age and gender differences. Eighty percent of the RGPs work more than 40 h per week. Face-to-face consultations accounts for little more than half of the regular working hours, though most of the working time is patient related. Nearly half of the RGPs have a position in the municipality in addition to their patient-related office work.

### Working hours per week

The total average working hours per week of 55.6 h including OOH primary care, and even the 49 regular working hours, is far above the recommended working time in Norway. According to the national regulatory framework for working hours (the Norwegian working environment act), the normal working hours must not exceed 40 h in 7 days [20]. A previous Norwegian study [17] found that the RGPs worked on average 48.6 working hours per week in 2014, including OOH primary care. The working hours per week in the previous study are, however, not directly comparable to our study, as the RGPs reported working hours in an average working week, while in our study the RGPs registered their working hours as precisely as possible each consecutive day during one specific week. In other countries, the working time per week is found to be 45 to 47 h per week in Dutch studies [21, 22], 50 in Germany [23], and 51 in Canada [24]. In a survey among general practitioners in 10 countries in 2015, the reported average working hours per week varied between 40 (Norway, Sweden, Australia, Canada) and 50 (Germany) [25]. The increase in reported working hours among RGPs in Norway from 40 in the 2015-study to 49 in our study in 2018 is considerable. The fast change in workload among RGPs in Norway may reflect the pressure to reduce access to secondary care, and the growing number of responsibilities that have been transferred from secondary to primary health care the last few years. Different organization of the health services may contribute to the variability in working hours between countries.

The gender difference in working hours was small in our study, as women work only one and a half hours less per week than men. In other countries there has been shown larger gender differences [22, 26–28]; examples are women working 8 h less than men among Dutch GPs [22], and 6 h less among English GPs [27]. A previous Norwegian study found that the increase in weekly working hours for RGPs from 2001 to 2008 was found mainly among women [16], which suggest a decreased gender difference in working hours among Norwegian RGPs. It has been asserted that feminisation of the profession is probably one of the reasons for the shortages of GPs and the decreased number of hours worked by GPs seen in some European countries [22]. This feminisation is also seen in Norway but does not seem to affect the regular working hours by Norwegian RGPs to the same extent. The proportion of female students at the Faculties of Medicine at the Norwegian Universities is now above 70% [29]. There is reason to be aware that this can also lead to changes in the desired workload among Norwegian RGPs in the future. We found that RGPs at around 60 years of age worked the highest number of hours per week, which is similar to the findings among Dutch GPs [22]. As the women were significantly younger than men, the difference among age groups in working hours per week may be explained by gender.

Self-employed RGPs worked significantly more hours per week than the RGPs with fixed salary, and the same is found among Dutch GPs [22]. While the difference was 7 h per week in Norway, the difference was as much as 14 h per week in the Dutch study. However, the group of RGPs with fixed salary is small and this kind of position is most often found in small municipalities with high OOH workload. Comparing regular working hours between the small and large municipalities may therefore be of less relevance.

Face-to-face consultations accounted for little more than half of the total working time among the RGPs. This is even less than found in another Norwegian study, which concluded that RGPs reduced the proportion of time spent on direct patient care from 73 to 69% in the period 1994–2014 [17]. In the 2015 survey performed in 10 countries, the “face-to-face-contact” with patients among RGPs in Norway was on average 70% of the total working time, and the proportion varied between 60 and 85% between countries [25]. Studies from UK, Portugal and USA have shown that direct patient care is between 61 and 67% of the total working time [30–32]. However, these numbers are not directly comparable due to variations in what was included in the concept of direct patient care (face-to-face only or including other patient-related work), and different methods of data collection (self-report versus external observation) [33]. The optimal proportion of time spent on direct patient care is not known. Good patient care also depends on tasks like writing record notes, referrals, certificates, administration and quality improvement. However, a decrease in direct patient care could potentially have negative implications for patient care and result in less satisfaction both among patient and doctors. The implications of decreased direct patient care and potential solutions to achieve good patient care should be further investigated.

### Additional activities

Only half of the RGPs had an additional position in the municipality or other professional activities. A study among Swiss GPs found that more than 90% were engaged in at least one activity beyond their in-office consultation [34]. We found that RGPs in smaller municipalities worked more hours in additional work in the municipality than RGPs in larger municipalities. This is supported by an Australian study, where rural GPs were twice as likely as urban GPs to work in municipality health settings and geriatric facilities [35]. In large municipalities, other doctors than the RGPs often are employed in for example nursing homes and OOH primary health care. In small municipalities, and thus among RGPs with small patient lists, such jobs are distributed among the RGPs themselves, thus increasing their work load on such tasks.

RGPs with large patient lists had more hours of patient-related work, but the number of hours did not increase linearly by number of patients on the list. We found less than a doubling in work time even if the list size was tripled from 600 patients to more than 1800. Several factors may explain this. Some RGPs are building up their practice from a new list starting with 0 patients, but temporarily help their colleagues and thus have more patient-related work than their own list size generates. Some have a younger population in their list and thereby less work per patient and some have older populations with more chronic diseases and time-consuming patients. Economic incentives have been studied earlier but have not been found to explain the variations [36–38]. Lastly, working style and effectiveness are quite individual [39]. Our results support that variations in working time cannot be explained by the list size alone.

Administration of office practice was not associated with list size. The administrative burden is equal even if the list size is short, as the same facilities are needed. The variation probably is explained by different participation in this kind of work. In a GP group practice, some will do more administration than others. Some practices will buy more external service for administration while others prefer to do it by themselves. And finally, the municipality performs administrative tasks on behalf of some RGPs.

### Study strengths and limitations

An important strength of this study is that we obtained measurement by using a survey to monitor working time of the different tasks, both clinical and others, of a large group of RGPs 1 week in real time, which should provide data of high reliability.

There are some limitations. The response rate was 39.8%, with a high risk of non-response bias. Compared to the overall RGP statistics in Norway 2017, our study included a higher proportion of women (49% vs 42%), persons between 30 and 39 years (32% vs 28%) and RGP specialists (68% vs 62%) [18]. The size of patient list was similar in the study and the RGP statistics. Thus, due to the relatively small differences, we assume that the study results are representative for the RGPs in Norway. Considering the task of recording a large number of variables 24 h for a full 7 days’ week, our response rate could be considered as very satisfactory.

The study was based on self-report, and there has been concern that reliance on provider self-report may yield results of low validity. The participants might have given answers in the direction they perceived were of interest, which then will have produced reporting bias [40]. The registration of working hours was performed in 1 week in January, and it is not known whether the measurement is representative for the RGPs working hours in general.

### Further research

There is a need for more knowledge about RGPs’ working time and the effect on their health, on the retention and recruitment of RGPs, as well as on the implications for quality of health service in primary care. More research should also be performed on factors found to be associated with working hours, like gender and size of municipality. In addition, more interventions should be performed in order to evaluate strategies for recruiting and retaining RGPs.

## Conclusions

The Norwegian RGPs have long working hours compared to recommended regular working hours in Norway. Women RGPs work almost as much as men. Just a little more than half of the working time is used on face-to-face consultations. There seems to be a trend of increasing the workload among Norwegian GPs, at the cost of direct patient contact. Further research should address identifying factors that can reduce long working hours.

## Additional file

Additional file 1: Questionnaire. (DOCX 46 kb)

## Data Availability

The datasets used during the current study are available from the corresponding author on reasonable request.

## Abbreviations

GP: General practitioner
RGP: Regular general practitioner
