# Supplementary material for: Workload in Norwegian general practice 2018 – an observational study
Source: BMC Health Serv Res. 2019 Jun 28;19:434. doi: 10.1186/s12913-019-4283-y (PMC6599272; doi:10.1186/s12913-019-4283-y)
Supplement: Supplementary file 1 — Questionnaire. (DOCX 46 kb) [file 12913_2019_4283_MOESM1_ESM.docx]

Workload in regular general practice (RGP)

National Center for Emergency Primary Health Care conducts the survey on behalf of the Norwegian Directorate of Health.

Practical information

Time estimated to complete the form is 2-8 minutes each day, depending on the variation in workload.

Start by fulfilling your RGP profile.

Time recording can start any weekday, but it is important that you complete the registration for seven consecutive days. We recommend that you fulfill the form every day after the end of working hours or the next morning if working late or in shift. If necessary, use the print-out of the list of tasks (see email invitation), the reimbursement claim and the appointment diary to support your memory. You are not supposed to fill out the day form while you work.

Exit the survey by clicking the cross at the top right of the window. Re-enter by using the link in the email. You will then enter the survey where you last exited. Move back and forth in the survey using the arrow keys. It is possible to adjust and add registrations until you submit the form the seventh day.

We do not need rigorous accuracy, but a good estimate of your activities during the day. Empty field represents activity not conducted this day. Every 24 hours starts from 08:00 am and ends the next morning at 07:59 am.

**Do you work as a GP full time or part time during participation of this survey?**

- Yes, in private RGP practice
- Yes, I’m a locum RGP
- No

|  |  |
| --- | --- |

If the respondent’s answer is No to the previous question, the survey ends and the following text appears:

Thank you for taking the time to respond. The survey is completed.

If the respondent’s answer is Yes to the previous question, the survey will proceed as follows

**1. Are you?**

- Male
- Female

**2. Your age**

- less than 30 years
- 30-34 years
- 35-39 years
- 40-44 years
- 45-49 years
- 50-54 years
- 55-59 years
- 60-64 years
- 65 years or more

**3. Are you a GP specialist?**

- Yes
- No

**4. For how many years have you been a GP?**

- 0 years
- 1 years
- 2 years
- 3 years
- 4 years
- 5 years
- 6 years
- 7 years
- 8 years
- 9 years
- 10 years
- 11 years
- 12 years
- 13 years
- 14 years
- 15 years
- 16 years
- 17 years
- 18 years

**5. For how many years have you worked as an RGP?**

- 0-2 years
- 3-5 years
- 6-10 years
- 11-15 years
- 16-25 years
- More than 25 years

**6. How many inhabitants are on your RGP list?**

- Less than 600
- 601-900
- 901-1200
- 1201-1500
- 1501-1800
- more 1800

**7. How many consultation days do you perform at your GP practice?**

- 0
- 1 day
- 1,5 days
- 2 days
- 2,5 day
- 3 days
- 3,5 day
- 4 days
- 4,5 day
- 5 days

**8. How many inhabitants live in the municipality where your RGP practice is based?**

- Less than 3000
- 3001-5000
- 5001-10 000
- 10 001-25 000
- 25 001-50 000
- 50 001-100 000
- More than 100 000

**9. What is the general distance in time from your GP practice to the nearest hospital with emergency services?**

- Less than 30 minutes
- 30 minutes - 1 hour
- 1-2 hours
- More than 2 hours

**10. What kind of RGP employment status do you have?**

- Self- employment
- Self- employment with municipality agreement on office, equipment and support personnel/ employees
- Fixed salary
- Fixed salary including all types of bonus agreements/schemes

For each of the categories in question 11 to 12 the respondent can choose between these answers for time spent:

Empty field

3 h 45 min (10 %)

7 h 30 min (20 %)

11 h 15 min (30 %)

15 h (40 %)

18 h 45 min (50 %)

22 h 30 min (60 %)

26 h 15 min (70 %)

30 h (80 %)

33 h 45 min (90 %)

37 h 30 min (100 %)

The fields are initially empty

**11. If you perform municipality related tasks, how many hours per week (percentage of employment) are you employed for this type of work?**

Kindly only answer on the tasks that are relevant to you. Empty fields are recorded as zero time used.

|  |  |
| --- | --- |
| Parent and child clinic | ▼ 3 h 45 min (10 %) ... 37 h 30 min (100 %) |
| Youth health services | ▼ 3 h 45 min (10 %) ... 37 h 30 min (100 %) |
| Nursing home/elder care | ▼ 3 h 45 min (10 %) ... 37 h 30 min (100 %) |
| Municipal emergency beds | ▼ 3 h 45 min (10 %) ... 37 h 30 min (100 %) |
| Fixed salary OOH-duty at day time | ▼ 3 h 45 min (10 %) ... 37 h 30 min (100 %) |
| Other patient related (prison etc.) | ▼ 3 h 45 min (10 %) ... 37 h 30 min (100 %) |
| Administrative position position (district medical officer, casualty clinic medical officer, infection control, adviser) | ▼ 3 h 45 min (10 %) ... 37 h 30 min (100 %) |

**12. What is your agreed time (percentage of employment) each week for other kind of work employments (or social security benefits)?**

Kindly only respond to the tasks that are relevant to you. Empty field recorded as zero time used.

|  |  |
| --- | --- |
| Research/teaching | ▼ 3 h 45 min (10 %) ... 37 h 30 min (100 %) |
| NAV (Norwegian Labour and Welfare Organisation), Military etc | ▼ 3 h 45 min (10 %) ... 37 h 30 min (100 %) |
| Retired, disability | ▼ 3 h 45 min (10 %) ... 37 h 30 min (100 %) |
| Occupational Health care | ▼ 3 h 45 min (10 %) ... 37 h 30 min (100 %) |
| Other, please describe in the field: | ▼ 3 h 45 min (10 %) ... 37 h 30 min (100 %) |

**24h registration for one week**

In the next section we kindly ask you to record how much time you have spent on different tasks each day within a week (Monday-Sunday). If necessary, use the print- out of the to-do list (see e-mail invitation), the 24-hour reimbursement claim and the appointment diary to support your memory.

Move back and forth in the survey and start recording for the current day. Please register for all seven days of the week. When you have register for one day, please close the website. The next day you can easily open the link to the questionnaire and continue where you stopped.

Kindly only answer for the tasks that are relevant to you. Empty fields recorded as zero time spent.

**Monday** 08:00 am-Tuesday 07:59 am
 
Please register how much time spent on different tasks from Monday 08:00 am to Tuesday 7:59 am.
Kindly only answer for the tasks that are relevant to you in Questions 1-8. Empty fields recorded as zero time spent. Remember to register any absence from practice under question 8. Even if you did not have a break, please answer to question 9.


If necessary, use the print-out of the to-do list (see e-mail invitation), the 24-hour reimbursement claim and appointment diary to support the memory.

For each of the categories in the questions for Monday the respondent has the following options for time spent:

Empty field

| 15 min  30 min  45 min  1 hour  1 h 15 min  1 h 30 min  1 h 45 min  2 hours  2 h 15 min  2 h 30 min  2 h 45 min  3 hours  3 h 15 min  3 h 30 min  3 h 45 min  4 hours  4 h 15 min  4 h 30 min  4 h 45 min  5 hours  5 h 15 min  5 h 30 min  5 h 45 min  6 hours | 6 h 15 min  6 h 30 min  6 h 45 min  7 hours  7 h 15 min  7 h 30 min  7 h 45 min  8 hours  8 h 15 min  8 h 30 min  8 h 45 min  9 hours  9 h 15 min  9 h 30 min  9 h 45 min  10 hours  10 h 15 min  10 h 30 min  10 h 45 min  11 hours  11 h 15 min  11 h 30 min  11 h 45 min  12 hours | 12 h 15 min  12 h 30 min  12 h 45 min  13 hours  13 h 15 min  13 h 30 min  13 h 45 min  14 hours  14 h 15 min  14 h 30 min  14 h 45 min  15 hours  15 h 15 min  15 h 30 min  15 h 45 min  16 hours  16 h 15 min  16 h 30 min  16 h 45 min  17 hours  17 h 15 min  17 h 30 min  17 h 45 min  18 hours | 18 h 15 min  18 h 30 min  18 h 45 min  19 hours  19 h 15 min  19 h 30 min  19 h 45 min  20 hours  20 h 15 min  20 h 30 min  20 h 45 min  21 hours  21 h 15 min  21 h 30 min  21 h 45 min  22 hours  22 h 15 min  22 h 30 min  22 h 45 min  23 hours  23 h 15 min  23 h 30 min  23 h 45 min  24 hours |
| --- | --- | --- | --- |

**1. RGP practice**

|  |  |
| --- | --- |
| Patient-related office practice /relatives present | ▼ 15 min ... 24 hours |
| Referrals, prescriptions, requisitions, discharge summary, test results and examinations, writing record notes (without patient present) | ▼ 15 min ... 24 hours |
| Certificates and declarations (without patient present) | ▼ 15 min ... 24 hours |
| Dialogue meetings, responsibility group,incl. any travel time. | ▼ 15 min ... 24 hours |
| Home visits incl. any travel time. | ▼ 15 min ... 24 hours |
| Administrative office practice (IT, accounting, meetings, HSE, procedures, purchasing) | ▼ 15 min ... 24 hours |
| Simple contacts, telephone and e-communication with patients  /relatives | ▼ 15 min ... 24 hours |
| Telephone and e-communication with collaborators (specialist health services, Pharmacy, Norwegian Labour and Welfare Organisation etc.) | ▼ 15 min ... 24 hours |
| E-consultations | ▼ 15 min ... 24 hours |

|  |  |
| --- | --- |
| **2. Municipality position/work** (parent and child clinic, nursing home, adm. position etc.) | ▼ 15 min ... 24 hours |
| **3. Other professional work** (research/teaching, Norwegian Labour and Welfare Organisation, Military, | ▼ 15 min ... 24 hours |

**4. OOH primary health care**

|  |  |
| --- | --- |
| Work at the OOH vlinic | ▼ 15 min ... 24 hours |
| OOH work on duty from home (incl. patient work) | ▼ 15 min ... 24 hours |
| Secondary on-call (all types, incl. intern on-call) | ▼ 15 min ... 24 hours |

**5. Various tasks**

|  |  |
| --- | --- |
| Union representative, municipality meetings | ▼ 15 min ... 24 hours |
| Supervision, teaching (students, others) | ▼ 15 min ... 24 hours |
| Self-education (reading, online courses etc.) | ▼ 15 min ... 24 hours |
| Courses, conferences, small groups, specialist meetings etc. | ▼ 15 min ... 24 hours |

**6. How much time of the previous question 1-5 (practice, referrals, declarations etc) relates to patients and their relatives with large and complex needs, given a total estimate for today?**

Total time can be more than the total workday, because some patients relates to several categories.

|  | |  |
| --- | --- | --- |
| Chronically ill | | ▼ 15 min ... 24 hours |
| Mental illnesses and drug addiction | | ▼ 15 min ... 24 hours |
| Frail elderly  Development disability and disabilities | | ▼ 15 min ... 24 hours  ▼ 15 min ... 24 hours |
|  |  |  |

**7. How much of the time in the previous question 1-5 (practice, referrals, declarations etc) relates to the following groups, given a total estimate for today?**

|  |  |
| --- | --- |
| Children and youths less than 25 years | ▼ 15 min ... 24 hours |
| Elderly more than 80 years | ▼ 15 min ... 24 hours |

|  |  |
| --- | --- |
| **8. Temporary absence this day (sick leave, leave, other reasons)** | ▼ 15 min ... 24 hours |

|  |  |
| --- | --- |
| **9. How much break (lunch- and rest periods) did you get during the work hour the last 24 hours?** ( | ▼ 15 min ... 24 hours |

At this point, you have passed the page for Monday.
You have now three options:

- Register a current day by scrolling back or forward. The survey organizes from Monday to Sunday. For example, if you start on Sunday, you have to scroll *back* to Monday.
- Pause the registration by closing the browser window. The responses will be automatically saved. The next day, open the link to the questionnaire and browse back or forward to the current day.
- Exit the survey if you have registered all current days, by scrolling to the last page and submitting.

Now the rest of the days follows until Sunday. After each day, a page with the following text will appear.

You have now passed the page for *(Tues-, Wednes-, Thurs-, Fri-, Satur-, Sun-)* day.

You have now three options:

- Register a current day by scrolling back (or forward). The survey organizes from Monday to Sunday. For example, if you start on Sunday, you have to scroll *back* to Monday.
- Pause the registration by closing the browser window. The responses will be automatically saved. The next day, open the link to the questionnaire and browse back or forward to the current day.
- Exit the survey if you have registered all current days, by scrolling to the last page and submitting.
